# Mechanisms of *Pseudomonas* spp. as plant growth promoting rhizobacteria: plant nutrition and biocontrol

**DOI:** 10.1007/s10482-026-02352-4

**Published:** 2026-06-08

**Authors:** Felix Satognon, Victor Akuku

**Affiliations:** 1 Department of Crop, Soil and Environmental Sciences, Auburn University, Auburn, AL USA; 2 Department of Soil and Crop Sciences, Texas A&M University, College Station, TX USA

**Keywords:** *Pseudomonas* spp., Plant growth promoting rhizobacteria, Biocontrol, Plant nutrition, Antibiotics

## Abstract

Increasing concerns over excessive agrochemical use, the emergence of resistant phytopathogens, and the impacts of climate variability on crop productivity have increased interest in plant growth promoting rhizobacteria (PGPR) as promising biological alternatives for sustainable crop production. *Pseudomonas* spp. are among the most extensively studied PGPR due to their remarkable metabolic diversity, strong rhizosphere colonization, and multifunctional roles in enhancing plant nutrition, improving stress tolerance, and suppressing plant diseases. These Gram-negative bacteria enhance plant growth through diverse direct and indirect mechanisms, including biological nitrogen fixation or stimulation of nitrogen fixation in co-existing diazotrophs, phosphate solubilization, siderophore-mediated iron acquisition, nutrient and niche competition with pathogens, secretion of antimicrobial metabolites, and activation of induced systemic resistance in plants. Recent advances in omics technologies have improved understanding of the mechanisms underlying *Pseudomonas* mediated plant nutrition and biocontrol. However, these insights remain fragmented across the literature, limiting a comprehensive understanding of Pseudomonas–plant interactions. This review synthesizes current knowledge on nutrient mobilization, siderophore activity, antimicrobial metabolite production, and induced plant defense responses associated with beneficial *Pseudomonas* spp. Emphasis is placed on how these mechanisms collectively enhance plant productivity, stress tolerance, and disease resistance, highlighting the potential of *Pseudomonas* spp. as sustainable biofertilizers and biopesticides.

## Introduction

The rhizosphere is the narrow zone of soil surrounding and directly influenced by plant roots (York et al. 2016). This dynamic region is shaped by rhizodeposition, which promotes the establishment of diverse microbial communities, including members of the genus *Pseudomonas* (Solomon et al. 2024). This specialized micro-environment is a cornerstone of nutrient cycling, essential for both environmental stability and the advancement of sustainable agriculture (Philippot et al. 2013). As a metabolically diverse genus within the class Gammaproteobacteria, *Pseudomonas* consists of Gram-negative bacteria ubiquitous in soil, aquatic systems, and various host organisms (Palleroni 2008). Their ecological success is driven by their abilities to utilize a vast array of organic substrates for energy and an inherent resilience to diverse antimicrobial compounds used in clinical and agricultural settings (Hesse et al. 2018). Furthermore, their capacity to produce a wide range of bioactive secondary metabolites (Hesse et al. 2018) underscores their physiological and genetic versatility, allowing them to flourish across myriad ecological niches (Sah and Singh 2016).

Within agricultural ecosystems, *Pseudomonas* species play a pivotal role in processes such as organic matter decomposition, nutrient recycling, and the mediation of plant–microbe interactions (Sah et al. 2021). In the rhizosphere, many species of *Pseudomonas* function as plant growth promoting rhizobacteria (PGPR), enhancing nutrient acquisition through mechanisms such as nitrogen (N) fixation, phosphate solubilization, and production of iron-scavenging siderophores (Alattas et al. 2024; Hakim et al. 2021; Panpatte et al. 2016). Furthermore, these bacteria are known to trigger induced systemic resistance (ISR), fortifying host plants against a broad spectrum of environmental stressors and pathogens (Alattas et al. 2024; Meena et al. 2020). However, the genus also includes important phytopathogens, highlighting the functional diversity of *Pseudomonas*. For instance, *Pseudomonas syringae* is a highly adaptable pathogen with an extensive host range, causing devastating infections in economically vital crops such as tomato (*Solanum lycopersicum*), tobacco (*Nicotiana tabacum*), and olive *(Olea europaea*) (Sah et al. 2021). This functional diversity, ranging from plant growth promotion to virulent pathogenicity, is a direct result of the metabolic versatility and genetic adaptability that define the genus.

Owing to their fast growth, strong root-colonizing ability, production of growth-enhancing metabolites, and resilience to abiotic stresses, *Pseudomonas* species are regarded as valuable tools in agricultural biotechnology. Experimental evidence supports the use of *Pseudomonas* PGPR strains to boost plant productivity. For example, the application of *P. fluorescens* significantly improved growth and yield in chickpea (*Cicer arietinum*) (Rokhzadi et al. 2008). Likewise, the beneficial *P. aeruginosa* strains isolated from sugarcane (*Saccharum officinarum*) tissues and rhizosphere notably enhanced both fresh and dry biomass (Mehmood et al. 2023). These studies emphasize the promising potential of selected *Pseudomonas* strains as biofertilizers and agents of sustainable crop enhancement. However, despite these promising applications as biofertilizers, a critical gap remains in the understanding of the precise mechanisms that govern these beneficial interactions with the host plant under fluctuating field conditions. To address this, the current review delves into the complex mechanistic strategies employed by *Pseudomonas* to optimize plant nutrition and provide effective biocontrol against recalcitrant phytopathogens.

## Nitrogen availability to plants

### Biological N fixation by *Pseudomonas* spp.

*Pseudomonas* species contribute to plant N availability through multiple interconnected mechanisms that operate at both cellular and community levels (Table 1). Certain members of the Pseudomonadaceae family, particularly diazotrophic strains (*Azotobacter vinelandii* AvOP, *Pseudomonas stutzeri* (A1501 and DSM4166), *Pseudomonas azotifigens* 6HT33bᵀ, and *Pseudomonas* sp. strain K1, *Pseudomonas protegens* CHA0), are capable of fixing atmospheric N (N_2_) into ammonium (NH_4_⁺), a plant-available form of N that enriches the rhizosphere and benefits both microbes and host plants (Mahmud et al. 2020; Yu et al. 2011; Wang et al. 2020). Biological N fixation (BNF) is the prokaryotic conversion of atmospheric N_2_ into ammonia (NH_3_), which is subsequently converted into NH4⁺ enabling plant growth in N-limited habitats (Rana et al. 2023). Although BNF is widely distributed among bacteria and archaea, it was long considered absent in. This process is enabled by the presence of a specialized N fixation island (NFI), which contains the core *nif* genes encoding the nitrogenase enzyme complex responsible for catalyzing N fixation (Leigh and Dodsworth 2007). For complete reduction of one N_2_ molecule, the Fe-protein delivers 8 electrons to the MoFe-protein, and each electron transfer step requires 2 ATP, implying a minimum of 16 ATP per molecule of N_2_ reduced (and typically additional energetic costs in vivo due to electron delivery and protective physiology) (Alleman and Peters 2023). Because nitrogenase metal cofactors and associated electron-transfer components are readily damaged by O_2_, diazotrophs either restrict N fixation to low-oxygen (microaerobic/anoxic) conditions or deploy oxygen-protection strategies (Robson and Postgate 1980). Although BNF is widely distributed among bacteria and archaea, it was long considered absent fromin the Pseudomonas genus until well-documented diazotrophic strains were characterized and sequenced, notably the root-associated strain *P. stutzeri* A1501 (Yan et al. 2008).

**Table 1:** Biological nitrogen fixation traits of *Pseudomonas* strains

Strains	Nitrogen-related functions
*P. stutzeri* A1501	Possesses a N fixation island (NFI) containing *nif* genes responsible for synthesizing nitrogenase, the enzyme complex that reduces atmospheric N_2_ into NH_4_⁺ (Ke et al. 2019)
*P. fluorescens*	Indirectly promotes N fixation by enhancing the activity of other N-fixing microbes (Zhang et al. 2018)
*P. putida*	Capable of atmospheric N fixation through nitrogenase activity; additionally plays a role in ammonification and denitrification, thus contributing to overall N turnover in soil (Shabayev 2010)
*P. aeruginosa* strain PGP	Carries *nif* genes for nitrogenase, enabling N fixation under low-oxygen (microaerophilic) conditions; exhibits tight regulation of N fixation gene expression (Roychowdhury et al. 2019)
*P. azotofixans*	A dedicated N fixer that efficiently converts atmospheric N into bioavailable forms using nitrogenase enzymes (Navarro‐Noya et al. 2013)
*P. protegens* Pf-5 X940	Enhances N fixation in plants through effective root colonization (Singh et al. 2024a, b)
*P. stutzeri* A15	Acts to boost N fixation, supporting plant N uptake (Pham et al. 2017)
*P. koreensis*	Contains a N fixation island, which includes genes essential for converting N_2_ to NH_4_⁺ (Li et al. 2017)
*P. pseudoflava*	Harbors a N fixation island, equipping it with the capacity to convert atmospheric N into plant-usable forms (Jenni et al. 1989)
Drought-tolerant *P. aeruginosa* strains (MK513745, MK513746, MK513747, MK513748, and MK513749)	Fix atmospheric N, thereby enhancing N availability and improving plant growth and yield under drought conditions (Uzma et al. 2022)
Engineering *P. protegens* Pf-5	Functions as both a biofertilizer and biocontrol agent by fixing atmospheric N and enhancing antifungal activity, thereby improving plant growth under nutrient-limited and pathogen-stressed conditions (Jing et al. 2020)

Comparative genomic analyses have revealed substantial variability in the structure, number, and regulation of *nif* genes among *Pseudomonas* species, reflecting evolutionary adaptation to diverse environmental conditions. Notably, complete genome sequencing of *P. stutzeri* DSM4166 identified genetic determinants associated with N fixation, denitrification, and rhizosphere competence, providing insight into the evolutionary acquisition of N-fixing traits in *Pseudomonas*, likely mediated by lateral gene transfer (Yu et al. 2011). Postgate and Kent (1987) demonstrated the successful horizontal transfer of *Klebsiella pneumoniae* nif genes into *P. putida* MT20-3, where the introduced genes were functionally expressed, resulting in detectable nitrogenase activity. This finding, together with reports of nodulation and N‑fixation genes moving from symbiotic to nonsymbiotic rhizobia via mobile genetic elements (Setten et al. 2013), highlight the broader potential for functional *nif* transfer across diverse bacterial hosts. This aligns with evidence that N‑fixation islands, a rare feature in *Pseudomonas*, have recently moved among strains and provide a genomic background capable of efficiently packaging and expressing the full *nif* complement, similar to the mobile N‑fixation and nodulation genes reported in rhizobia (Yang et al. 2018a, b).

**Fig. 1:**
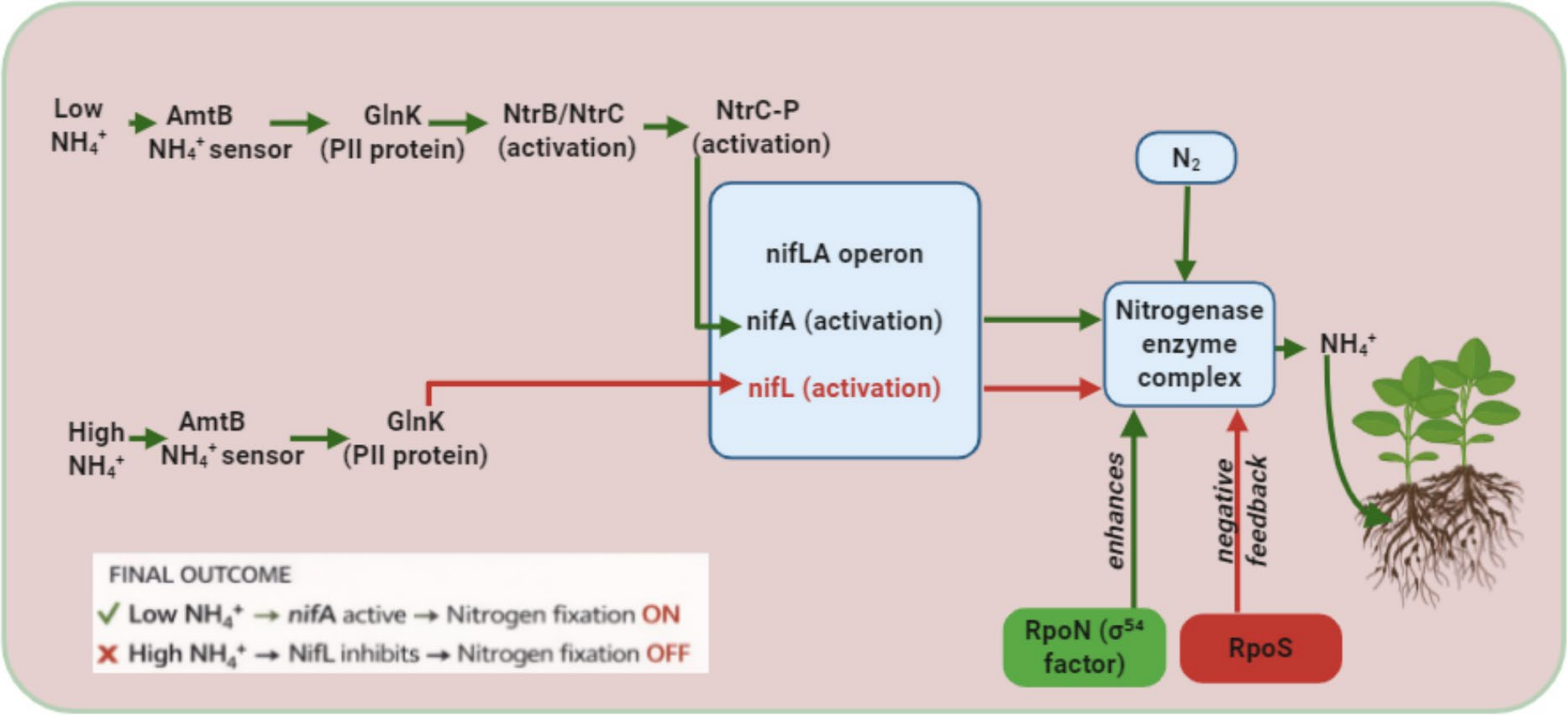
Molecular mechanism of N fixation by *Pseudomonas* spp.

Biological N fixation actitivity of *Pseudomonas* spp is highly regulated. While some species exhibit constitutive expression of N fixation genes as an adaptation to chronically N-deficient soils, others tightly regulate *nif* expression in response to external N availability (Anderson 1955). For example, *P. stutzeri* A1501, which is now recognized as a bona fide N-fixing bacterium harbors the largest known contiguous NFI (49 kb), organized into 11 operons under the control of the master regulator *nifA* and the σ^54^ sigma factor, ensuring efficient N fixation under fluctuating rhizosphere conditions (Desnoues et al. 2003). In *P. stutzeri* A1501, N fixation is controlled by the AmtB–GlnK–NtrBC signaling cascade, which modulates the nifLA operon (Fig. 1). Under N-limited conditions, *nifA* is activated via *NtrC* and *GlnK*; conversely, in NH_4_⁺-rich environments, *GlnK* mediates repression through *NifL*. Evidence for this regulatory hierarchy is seen in glnK-deficient mutants, where constitutive *nifA* expression restores nitrogenase activity even in the presence of NH_4_⁺, underscoring GlnK’s central role in NH_4_⁺ sensing (Sanow et al. 2023). Furthermore, N fixation is integrated into the broader Rpo/Gac/Rsm signaling network, where RpoN co-activates biofilm (pslA) and N fixation (*nifA*) genes, while RpoS provides negative feedback to fine-tune these processes in response to nutrient availability (Shang et al. 2021).

Given increasing concerns over the excessive use of inorganic fertilizers, these mechanisms, although largely demonstrated under controlled laboratory conditions, hold strong potential for real-world agricultural applications aimed at reducing dependence on synthetic N inputs. Their practical relevance is supported by studies reporting significant yield and N gains. For instance, inoculation of sugarcane (*Saccharum officinarum*; GT11 and G × B9) with *P. koreensis* CY4 and *P. entomophila* CN11 significantly increased N concentration and enrichment in plant tissues, particularly in stems and leaves (Singh et al. 2024a, b), confirming their capacity to fix atmospheric N and enhance growth under N-limited conditions. Similarly, Fox et al. (2016) reported that inoculating maize (*Zea mays*) and wheat (*Triticum aestivum*) with *P. protegens* Pf-5 X940 increased N content and biomass in both vegetative and reproductive tissues. ^15^N analysis in the same study further confirmed substantial atmospheric N uptake, indicating active root-associated N fixation. However, translating these promising results to field conditions remains challenging. Environmental variability, such as fluctuations in soil moisture, temperature, and pH, can strongly influence microbial survival and nitrogenase activity (Sun et al. 2019; Tiemann and Billings 2011). Competition with native soil microbiota often limits the establishment and persistence of introduced strains (Trabelsi and Mhamdi 2013). Additionally, oxygen sensitivity of nitrogenase, inconsistent carbon supply from plant roots, and variability in soil nitrogen levels can suppress biological N fixation in situ (Setten et al. 2013). Scaling up *Pseudomonas* spp inoculant production, ensuring formulation stability, and achieving consistent field performance across diverse agroecosystems also remain significant barriers to widespread adoption.

### Stimulation of N fixation in co-existing diazotrophs and enhancement of legume nodulation

*Pseudomonas* spp. also indirectly enhance N availability in the rhizosphere by stimulating biological N fixation in neighboring microbial communities. For example, *P. fluorescens* F113 produces secondary metabolite 2,4-diacetylphloroglucinol (DAPG), which induces *nif* gene expression in *Azospirillum brasilense* S245 (Combes-Meynet et al. 2011). Co-inoculation of rice (*Oryza sativa*) with *A. brasilense* and *P. fluorescens* increased grain yield by 37% per unit of N (Turino Mattos et al. 2023). In addition to influencing free-living diazotrophs, *Pseudomonas* spp such as *P. fluorescens* was reported to enhance nodulation in legumes, including chickpea (*Cicer arietinum*) (Kaur et al. 2015). This stimulation increases symbiotic N fixation by rhizobia, thereby improving plant N nutrition (Nagpal et al. 2021; Sanow et al. 2023). These effects may be mediated through multiple complementary mechanisms, including phytohormone (e.g., indole-3-acetic acid, IAA) production that stimulates root hair development and infection sites (Patten and Glick 2002), improved rhizobial signaling and colonization (Dardanelli et al. 2010), enzymatic facilitation of root infection (e.g., cellulase production) (Egamberdieva et al. 2010), and stress alleviation through 1-aminocyclopropane-1-carboxylate deaminase (ACC deaminase) activity, which lowers ethylene levels and promotes nodulation (Nascimento et al. 2016; Satognon et al. 2026). Collectively, these processes underscore the multifunctional role of *Pseudomonas* in plant–microbe dynamics. Flores-Duarte et al. (2022) found that co-inoculation of alfafa (*Medicago sativa*) with rhizobia and *Pseudomonas* increased plant biomass up to 1.5-fold and number of nodules up to twofold compared to rhizobia alone. Similarly, the co-inoculation of *Rhizobium* and *Enterobacter* strains with *Pseudomonas* sp. SV23 increased nodule formation in fava bean (*Vicia faba*) (Fatnassi et al. 2015). In soybean (*Glycine max*), co-inoculation with *Bradyrhizobium* strains (*B. diazoefficiens* USDA110 or *B. ottawaense* SG09) and *Pseudomonas* PGPB (OFT2 and OFT5) significantly improved plant growth, nodulation, and N_2_ fixation, with increases of up to 81% relative to single inoculations (Win et al. 2023). Comparable effects have been reported in fodder galega (*Galega orientalis*), where *P. trivialis* enhanced nodulation and plant N content (Egamberdieva et al. 2010), and in common bean (*Phaseolus vulgaris*), where co-inoculation with *Rhizobium pisi* and *P. monteilii* improved nodulation, growth, and yield across contrasting soil types, with genotype-specific responses (Sánchez et al. 2014). However, previous research also reported that co-inoculation of *Bradyrhizobium japonicum* A1017 with *P. brassicacearum* WCS365 reduced nodule numbers in soybean (*Glycine max*) (Chebotar et al. 2001), highlighting that *Pseudomonas* effects on nodulation are strain- and host-dependent (Yu et al. 2025).

While rare, specific *Pseudomonas* isolates possess the atypical capability to induce nodulation independently of traditional rhizobia. For example, *Pseudomonas* sp. Ch10048 was found to nodulate *Robinia pseudoacacia*, harboring a *nodA* gene closely related to that of *Mesorhizobium loti* (Shiraishi et al. 2010). Similarly, *Pseudomonas* sp. GLU4 carries *nodC* and induces non-fixing nodules on alfalfa, despite the absence of *nodA* and *nifH* genes. This further confirms a partial acquisition of symbiotic genetic elements via horizontal gene transfer (Wigley et al. 2017). In addition, *Pseudomonas* strains are frequently detected as nodule endophytes coexisting with primary rhizobia (Yu et al. 2025). Although most of these strains cannot independently initiate nodulation, they can substantially influence symbiotic N fixation efficiency. In *Lotus burttii*, inoculation of *Pseudomonas* spp with *Mesorhizobium* spp reduced the frequency of ineffective nodules, indicating that these endophytes can modulate symbiotic outcomes depending on the specific host-rhizobia partnership (Crosbie et al. 2022). However, the exact mode of entry for these non-nodulating strains remains a subject of investigation, with proposed mechanisms including "crack entry" via epidermal lesions or "hitchhiking" within infection threads initiated by symbiotic rhizobia (Yu et al. 2025).

## Phosphorus solubilization by *Pseudomonas* spp.

### Production of organic acids

**Fig. 2:**
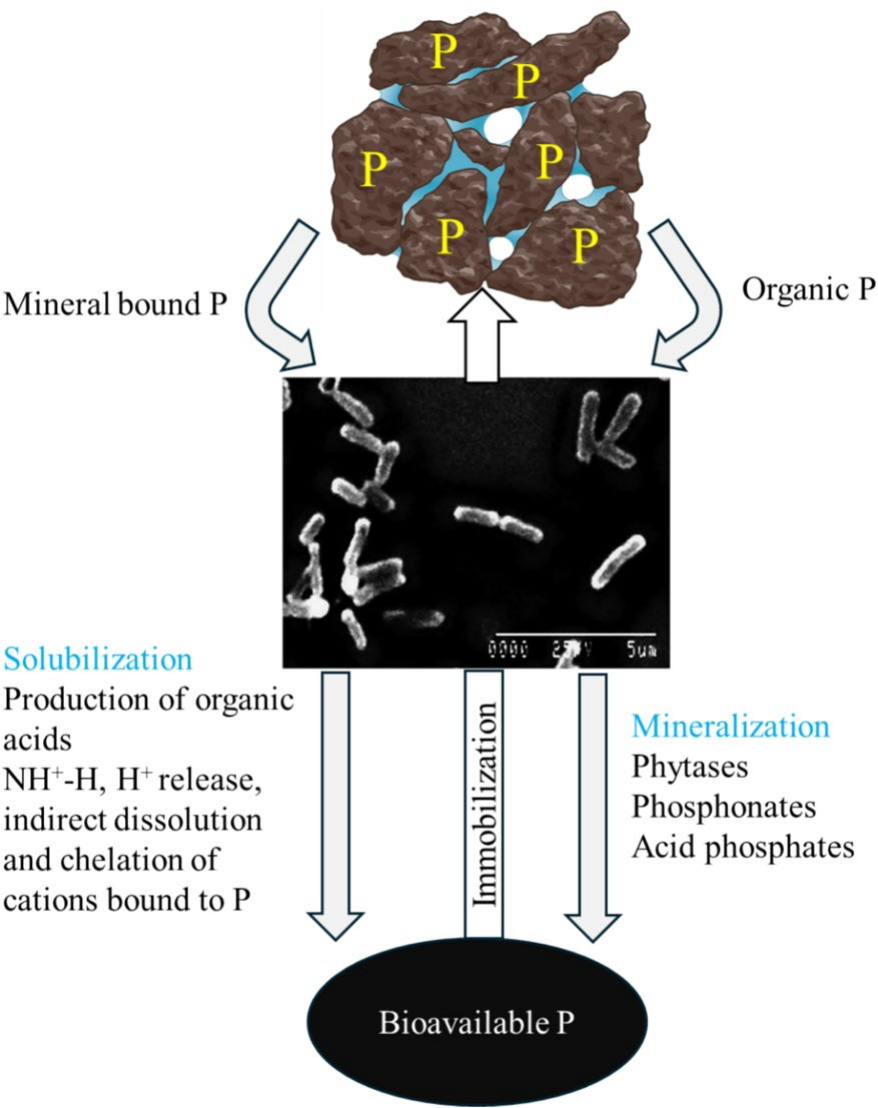
Mechanism of phosphorus solubilization and mineralization to facilitate increased P availability for plant uptake within the rhizosphere

Phosphorus (P) is a critical macronutrient for plant development, yet it often exists in insoluble forms that are unavailable for uptake (Akuku and Satognon 2025). The primary mechanism by which *Pseudomonas* species solubilize inorganic phosphate involves the exudation of organic acids (Fig. 2) (Miller et al. 2010). The release of these low-molecular-weight organic acids into the rhizosphere reduces the localized pH, facilitating the dissolution of insoluble phosphate minerals (e.g., calcium, aluminum, and iron phosphates) (Kaur et al. 2016). This mobilization occurs through proton release, chelation of metal cations, or ligand exchange, which effectively liberates orthophosphate ions from mineral surfaces (Rodríguez and Fraga 1999). The biosynthesis of these organic acids is driven by extracellular oxidative metabolism of glucose. This metabolic pathway is mediated by key periplasmic enzymes, most notably glucose dehydrogenase (Gcd) and gluconate dehydrogenase (Gad) (Gnyp et al. 2014). The specific organic acids produced by various *Pseudomonas* strains, which vary in their solubilization efficiency, are summarized in Table 2. While organic acid exudation represents a major route for mobilizing mineral‑bound phosphorus, many *Pseudomonas* strains complement this strategy with enzymatic mechanisms that target organic P pools.

**Table 2:** Organic acids produced by *Pseudomonas* strains

Organic acid produced	Predominant *Pseudomonas* spp.
Gluconic acid	*P. fluorescens* B16 (Goldstein and Liu 1987) *Pseudomonas* spp. (Li et al. 2023)
Formic acid, ascorbic acid, acetic acid, citric acid, and succinic acid	*Pseudomonas* sp. W134 (Li et al. 2023; Wang et al. 2022)
Acetic acid, pyruvic acid	*P. fluorescens* ATCC 13525 (Patel et al. 2008)
Gluconic acid, tartaric acid, citric acid, maleic acid and fumaric acid	*P. fluorescens* JW-JS1 (Hu et al. 2024)
Formic acid, Butyrate, and Propanedioic acid	*Pseudomonas* sp. PSB12 (Chen et al. 2016)
2-Ketogluconic acid	*P. cepacia* E37, *P.s plecoglossicida* (Wang et al. 2024)

### Enzyme activities

*Pseudomonas* species can also solubilize soil P primarily through the secretion of extracellular enzymes, particularly phosphatases, which mineralize both organic and mineral P compounds in soil (Mayer et al. 2025). This enzymatic transformation is critical for converting recalcitrant organic P fractions into bioavailable orthophosphate. Numerous studies have demonstrated that *Pseudomonas* spp. synthesize significant quantities of both acid and alkaline phosphatases, allowing them to remain active across a wide pH range (Dejsirilert et al. 1989; Liu et al. 2016; Lidbury et al. 2021, 2022). For example, Lidbury et al. (2022) identified a widely distributed, phosphate-insensitive alkaline phosphatase (PafA). Unlike many phosphatases that are inhibited by high concentrations of their end-product, PafA remains active across diverse environmental conditions, providing a continuous release of orthophosphate from phosphomonoesters in soil organic matter. This trait enables *Pseudomonas* to sustain P mineralization even in nutrient-fluctuating soil niches. Furthermore, Li et al. (2023) highlighted the role of *Pseudomonas* in mobilizing legacy phosphorus by catalyzing the transformation of accumulated mineral P pools into plant-available forms. Collectively, these findings underscore the ecological significance of *Pseudomonas* spp. as key drivers of the P cycle, ensuring sustained P availability through robust enzymatic production (Mayer et al. 2025; Lidbury et al. 2022). *Pseudomonas* strains reported to be more effective in P solubilization are summarized in Table 3.

**Table 3:** Examples of *Pseudomonas* strains and their mechanisms for solubilizing phosphorus

Strains	Phosphorus solubilization mechanisms
*P. aeruginosa*	Produces pyoverdine-type siderophores and excretes organic acids that aid in phosphate solubilization (Song et al. 2024; Lozano-González et al. 2023)
*P. kilonensis*	Utilizes both siderophore secretion and organic acid release to mobilize insoluble P forms (Safari et al. 2020)
*P. putida*	Solubilizes phosphate through the production of gluconic and 2-ketogluconic acids, siderophores, and phosphatase enzymes (Singh et al. 2024a, b; Song et al. 2024; Sah et al. 2021)
*P. extremaustralis* (PeCHP-2, -3)	Engages in P solubilization by releasing both acidic and alkaline phosphatases along with organic acids (Mayer et al. 2025)
*P. fluorescens* (CB501, CD511, CE509)	Facilitates phosphate solubilization via organic acid excretion, soil pH reduction, and phosphatase activity (Joshi et al. 2014)
*P. monteilii* (JAS-25)	Increases P availability by producing siderophores and secreting organic acids (Srivastava et al. 2022)

## Siderophore production

Siderophores are specialized, high-affinity iron-chelating molecules secreted by microorganisms including *Pseudomonas* spp to acquire essential iron in environments where it is scarce (De Serrano et al. 2016). These small molecules, usually between 500 and 1500 Daltons in size, show a high affinity for binding iron (III) (Hider and Kong 2010). Among these, *P. fluorescens* strains produce pyoverdines (PVD) and pseudobactins, which serve as primary chelators critical for ecological fitness (Hannauer et al. 2012). The genetic architecture governing these molecules reveals a conserved yet versatile system. For instance, *P. fluorescens* SBW25 carries at least 31 genes across seven genomic regions involved in pyoverdine biosynthesis and uptake, while in *P. putida* WCS358, at least 15 genes distributed across five clusters, including the non-ribosomal peptide synthetase (NRPS) gene *ppsD*, coordinate the assembly of pseudobactin 358 (Moon et al. 2008; Devescovi et al. 2001; Leong et al. 1991). This biosynthetic process is initiated in the cytoplasm via nonribosomal peptide synthetase (NRPS) and completed within the periplasm, after which the mature siderophore is exported into the extracellular milieu by the PvdRT-OpmQ ATP-dependent efflux system (Bonneau et al. 2020; Schalk et al. 2020). Once in the extracellular environment, the siderophore chelates ferric iron (Fe^3+^) with high specificity. The resulting complex is recognized and transported across the bacterial outer membrane by TonB-dependent receptors, such as FpvAI and FpvB, utilizing energy provided by the TonB-ExbB-ExbD inner membrane complex (Hannauer et al. 2012). In the periplasm, the complex is captured by the binding proteins FpvC and FpvF. Unlike other siderophores that undergo chemical degradation to release their cargo, *Pseudomonas* employs a sophisticated recycling mechanism. Iron is liberated from the pyoverdine through enzymatic reduction of Fe^3+^ to Fe^2+^ by the inner membrane reductase FpvG (Miller et al. 2010). Because Fe^2+^ possesses a significantly lower affinity for the ligand, it dissociates and is transported into the cytoplasm via the ABC transporter FpvDE (Brillet et al. 2012). Following dissociation, the iron-free (apo) form is stabilized by FpvF and recycled to the extracellular environment via the PvdRT-OpmQ pump to resume scavenging (Ganne et al. 2017; Schalk et al. 2020).

The regulation of these pathways is tightly governed by iron availability and environmental stressors. While many species follow a ferric uptake regulator (Fur) dependent repression model, some strains utilize unique mechanisms, such as the an extracytoplasmic family (ECF) sigma factor FpvI in *P. fluorescens*, which activates *fpvR* to modulate iron uptake (Moon et al. 2008). In the rhizosphere, this process increases iron availability and can indirectly improve plant iron acquisition, particularly in calcareous and alkaline soils where iron solubility is limited (Colombo et al. 2014). Beyond nutrient acquisition, siderophore production facilitates abiotic stress mitigation. Metal-tolerant strains, such as *P. fluorescens* PGPR-7, maintain siderophore and ACC deaminase production under cadmium stress, effectively reducing toxic metal uptake and improving plant physiological performance (Syed et al. 2023). Furthermore, siderophores contribute to plant adaptation in degraded soils by promoting iron mobilization and overall microbial competitiveness (Singh et al. 2022). In addition to their role in iron homeostasis, *Pseudomonas* siderophores are essential drivers of indirect P solubilization. By chelating metal cations such as Fe^3+^, siderophores disrupt insoluble phosphate complexes in the soil, releasing bioavailable orthophosphate (PO_4_^3−^) for plant uptake (Lozano-González et al. 2023; Wang et al. 2022). This dual functionality as both iron carriers and P mobilizers, combined with the secretion of organic acids and phosphatases, makes *Pseudomonas* an ideal candidate for biofertilizer development (Lidbury et al. 2022; Ou et al. 2022). Ultimately, these multifaceted mechanisms enhance environmental sustainability by reducing agricultural dependence on synthetic chemical fertilizers (Lidbury et al. 2021; Ou et al. 2022; Sah et al. 2021).

## Biocontrol potential of *Pseudomonas* spp.

*Pseudomonas* spp. are recognized as potent biocontrol agents (BCAs) due to their metabolic versatility, which includes the production of diverse antibiotics, promotion of plant growth, enhancement of nutrient uptake, and degradation of soil toxins (Ramadan et al. 2016). Their ubiquity in agricultural soils and efficacy against soilborne pathogens make them cornerstone components of biological control strategies. The biocontrol activity of *Pseudomonas* spp. generally operates through three primary mechanisms: (1) competitive exclusion via nutrient and niche competition, (2) direct antagonism through antibiosis, and (3) induction of systemic resistance (ISR) in host plants (Table 4) (Santoyo et al. 2012).

**Table 4:** Representative *Pseudomonas* strains and their biocontrol mechanisms

Strains	Target pathogens	Biocontrol-related traits
*P. aeruginosa* 7NSK2	Tomato–*Pythium* spp.	Produces iron-regulated antibiotics, induces systemic resistance (ISR), and synthesizes antimicrobial metabolites (Wensing et al. 2010)
*P. aeruginosa* F2	Wheat–*Fusarium oxysporum* and* Rhizoctonia solani*	secretes biocontrol siderophores that reduce *Fusarium oxysporum* and *Rhizoctonia solani* infections, while also boosting wheat plant growth (Abo-Zaid et al. 2023)
*P. fluorescens* JY3	Wheat–*Fusarium oxysporum* and* Rhizoctonia solani*	Produces high-efficiency siderophores that suppress these soil-borne pathogens and enhance plant biomass (Abo-Zaid et al. 2023)
*Pseudomonas* strains I-9 and I-22	*Aspergillus flavus*, *Fusarium graminearum*, *Penicillium italicum*, *Penicillium expansum*, *Phytophthora infestans*	Both strains produce hydrogen cyanide (HCN); strain I-9 also produces siderophores for iron acquisition and pathogen inhibition (Ganbat et al. 2025)
*P. brassicacearum* Q8r1-96	Wheat–*Gaeumannomyces graminis* var. *tritici*	Effective in root colonization, antibiotic production, and employs specialized secretion systems (Yang et al. 2018a, b)
*P. marginalis* ORh26	Sugar beet (*Beta vulgaris*)–*P. syringae* pv. *aptata* P21	Use T3SS to stimulate ISR in sugar beet
*P. chlororaphis* PCL1391	Tomato–*Fusarium oxysporum*	Known for strong root colonization and production of antifungal antibiotics ((Lugtenberg and Girard 2013)
*P. chlororaphis* O6	Tobacco–*Erwinia carotovora*; Cucumber–*Corynespora cassiicola*	Produces antiviral peptides, synthesizes 2R,3R-butanediol, an unidentified nematicidal compound, and induces ISR (Mercado-Blanco 2015)
*P. chlororaphis* 30–84	Wheat–*Gaeumannomyces graminis* var. *tritici*	Produces antifungal antibiotics for disease suppression (Mercado-Blanco 2015)
*P. aeruginosa* FG106	Tomato, Potato (*Solanum tuberosum*), Taro (*Colocasia esculenta*), and Strawberry (*Fragaria* × *ananassa*)–*Alternaria alternata, Botrytis cinerea, Clavibacter michiganensis* subsp*. michiganensis, Phytophthora colocasiae, P. infestans, Rhizoctonia solani,* and *Xanthomonas euvesicatoria pv. perforans*	Secretes lipases and proteases, induces high phosphate solubilization, produces siderophores, ammonia, indole acetic acid (IAA), and hydrogen cyanide (HCN) and forms biofilms that promote plant growth and facilitate biocontrol (Ghadamgahi et al. 2022)
*P. fluorescens* CHA0	Broad-spectrum–fungal pathogens, nematodes, TNV in tobacco	Produces siderophores, lytic enzymes, various antibiotics, and triggers ISR (Mercado-Blanco 2015)
*P. fluorescens* F113	Potato–*Erwinia carotovora* subsp. *atroseptica*	Known for antibiotic synthesis against bacterial pathogens (Abd-El-Khair 2019)
*P. aeruginosa* strain 91	Banana (*Musa* spp.)–*Fusarium oxysporum* f. sp. *cubense* (Foc)	Secretes cell wall hydrolytic enzymes and bioactive compounds while effectively colonizes root tissues to promote growth and suppress wilt (Xie et al. 2023)
*P. fluorescens* PCL1751	Tomato–*F. oxysporum* f. sp. *radicis-lycopersici*	Competes efficiently for colonization sites and nutrient resources (Miftakhov et al. 2022)
*P. putida* PCL1760	Tomato–*F. oxysporum* f. sp. *radicis-lycopersici*	Displays niche and nutrient competition, similar to PCL1751 (Miftakhov et al. 2022)
*P. fluorescens* Pf29Arp	Wheat–*G. graminis* var. *tritici*	Activates host defense responses, contributing to biocontrol (Marchi et al. 2013)
*P. fluorescens* PICF7	Olive (*Olea europaea*)–*Verticillium dahliae*	Functions as a root endophyte and triggers plant defense mechanisms (Gómez-Lama Cabanás et al. 2014)
*P. fluorescens* Q2-87	Wheat–*G. graminis* var. *tritici*	Synthesizes antibiotics and induces systemic resistance (Maketon et al. 2012)
*P. fluorescens* WCS374	Radish (*Raphanus sativus*)–*F. oxysporum* f. sp. *raphani*	Produces multiple siderophores, lipopolysaccharides, competes for iron (Fe^3^⁺), and induces ISR (Djavaheri et al. 2012)
*P. fluorescens* WCS417	Radish–*F. oxysporum* f. sp. *raphani*; *Arabidopsis thaliana*–*P. syringae* pv. *tomato*	Involved in ISR, synthesis of lipopolysaccharides, and iron-regulated metabolites (Prashar et al. 2013)
*P. pseudoalcaligenes* AVO110	Avocado (*Persea americana*)–*Rosellinia necatrix*	Competes efficiently for nutrients and root colonization niches (Pliego et al. 2019)
*P. protegens* Pf-5	Broad-spectrum–soilborne pathogens (fungi, oomycetes, bacteria)	Produces a wide array of antibiotics for broad-spectrum disease control (De la Vega-Camarillo et al. 2024)

### Rhizosphere colonization and niche competence

The rhizosphere is a hotspot of intense microbial competition for nutrients and space. Pseudomonas spp. excel in this environment, particularly under nutrient-limited conditions, facilitated by specialized traits such as the production of lipopolysaccharides (Raymond et al. 2002). Strains like *P. fluorescens* WCS417Rr exhibit robust root colonization and endosymbiotic tendencies, driven by the structural variability of O-antigen polysaccharides that allow for niche-specific adaptation (Raymond et al. 2002). Furthermore, motility, chemotaxis, and efficient carbon utilization are critical for securing spatial dominance and suppressing pathogens (Santoyo et al. 2021). This colonization capability is further bolstered by genomic plasticity, including recombinase-driven rearrangements (Santoyo et al. 2012). In iron-limited soils, *Pseudomonas* spp. secrete siderophores, high-affinity iron chelators, that sequester iron, thereby starving competing pathogens (Panpatte et al. 2016). Siderophores play a central role in boosting the survival and overall fitness of microbial communities, and also strengthen the effectiveness of biocontrol organisms both directly and indirectly (Alattas et al. 2024). Research shows that siderophores produced by *P. fluorescens* strain Mst 8.2 significantly suppressed *Rhizoctonia solani*, leading to a 70% reduction in wheat disease (Gull and Hafeez 2012). Likewise, siderophores from *P. fluorescens* Lp1 have been used to curb several fungal pathogens, including *Aspergillus flavus*, *Curvularia* species, and *Fusarium* species (Kanimozhi and Perinbam 2011). *Pseudomonas* strain ZUM80 produces pseudobactin, a siderophore with significant inhibitory effects on pathogens (Wensing et al. 2010). Although these suppressive effects are closely linked to rhizosphere competence, additional mechanisms likely contribute to the overall pathogen suppression capacity of *Pseudomonas* spp.

### Antibiotic and antifungal compound synthesis

Disease‑suppressive soils are often enriched with *Pseudomonas* spp. capable of producing a diverse arsenal of antifungal secondary metabolites (Mehmood et al. 2023; Azeem et al. 2022). Among these, 2,4‑diacetylphloroglucinol (DAPG/Phl) is one of the most extensively characterized determinants of soil suppressiveness (Suresh et al. 2022). DAPG‑producing *Pseudomonas* strains are reported to effectively control several root diseases, including tobacco black root rot, and their activity is tightly linked to rhizosphere colonization (O'sullivan and O'Gara 1992). Mendes et al. (2013) obsereved that Phl is actively synthesized on wheat roots in suppressive soils but is largely absent in non‑suppressive counterparts, underscoring its ecological relevance. Similar patterns have been observed in maize, where rhizosphere communities harbor significantly higher abundances of Phl biosynthetic genes compared to bulk soil (Picard et al. 2000). Beyond DAPG, many *Pseudomonas* biocontrol agents rely on a broader suite of antifungal metabolites, including pyoluteorin, pyrrolnitrin, and phenazines, that collectively inhibit pathogen growth and modulate microbial community structure (Lee et al. 2022). A large-scale survey of 700 *P. fluorescens* isolates in Uruguay identified several strains (UP61, UP143, and UP148) with strong in vivo activity against *Pythium ultimum* and *Rhizoctonia solani*. Notably, these strains did not disrupt symbiotic N fixation in legumes such as alfalfa, highlighting their compatibility with beneficial plant–microbe interactions (Höfte and Altier 2010).

### Induction of systemic resistance (ISR)

Induced systemic resistance (ISR) is a central mechanism by which *Pseudomonas* spp. enhance plant immunity at sites distant from the point of colonization (Yu et al. 2022). The signaling pathways underlying *Pseudomonas*-mediated ISR are mechanistically diverse. Several strains, including *P. fluorescens* EP1 and *P. putida* 5–48, have been shown to activate ISR in diverse hosts such as sugarcane and oak, conferring protection against aggressive fungal pathogens including *Colletotrichum falcatum* and *Ceratocystis fagacearum* (Mercado-Blanco 2015). In addition to direct microbe–plant interactions, volatile organic compounds (VOCs) such as hydrogen cyanide (HCN), dimethyl disulfide, 2,3-butanediol, and 1-undecene play a pivotal signaling role (Raza et al. 2016). For example, 2,3-butanediol produced by *P. chlororaphis* O6 have been reported to promote plant growth and triggers systemic immunity in tobacco (Cho et al. 2008). Montes-Osuna et al. (2022) found that *Pseudomonas* PICF6 and PICF7 suppress soil‑borne pathogens largely through a diverse, pathogen‑responsive volatilome composed of antifungal VOCs such as 1‑undecene, (methyldisulfanyl) methane, 1‑decene, and the induced compound 4‑methyl‑2,6‑bis(2‑methyl‑2‑propanyl)phenol, along with growth‑promoting volatiles like tridecane, enabling targeted inhibition of *Verticillium* spp. and potential broader benefits to olive plants.

Unlike Bacillus-induced ISR which typically depends on jasmonic acid (JA) and ethylene signaling, some *P. aeruginosa* strains activate salicylic acid (SA)-dependent defenses by synthesizing SA directly in the rhizosphere (Santoyo et al. 2012). Recent work has revealed a sophisticated defense trade-off governed by these hormonal pathways. Haney et al. (2018) demonstrated that closely related rhizosphere *Pseudomonas* strains can induce ISR, systemic susceptibility (ISS), or remain neutral toward foliar pathogens such as *Pseudomonas syringae* pv. *tomato* DC3000. For example, *Pseudomonas* sp. CH267 shifts the JA/SA balance toward JA-mediated herbivore defenses, enhancing resistance to *Trichoplusia ni*, but at the cost of SA-dependent antibacterial immunity. In contrast, the ISR-inducing strain *Pseudomonas* sp. WCS417 overrides the typical JA/SA antagonism and primes plants for both defense pathways simultaneously (Haney et al. 2018).

In the wake of expanding the mechanistic framework of ISR, recent findings have uncovered a novel beneficial role for the Type III Secretion System (T3SS) (Nedeljković et al. 2025; Zboralski et al. 2022). Traditionally associated with pathogenicity, the T3SS of *P. marginalis* ORh26 was shown to be essential for ISR induction in sugar beet against *P. syringae* pv. *aptata*. ORh26 activated peroxidase and phenylalanine ammonia-lyase and upregulated key defense regulators such as NPR1 and MYC2, whereas T3SS-deficient mutants failed to elicit these responses (Nedeljković et al. 2025). This discovery highlights a previously unrecognized function of T3SS in beneficial plant–microbe interactions and underscores the complexity of ISR signaling. Taken together, these findings illustrate that the biocontrol efficacy of *Pseudomonas* spp. is not the result of a single trait, but rather a coordinated integration of aggressive niche competition, a diverse metabolic arsenal, and sophisticated manipulation of host immunity. As the understanding of these molecular dialogues deepens, particularly regarding the surprising roles of ‘pathogenic’ machinery like the T3SS, it becomes clear that these bacteria are precision-engineered by evolution to serve as sustainable alternatives to synthetic agrochemicals in modern agriculture.

## Conclusion and future perspectives

*Pseudomonas* spp. represent one of the most metabolically versatile and functionally diverse groups of PGPR, contributing significantly to plant nutrition, stress tolerance, and disease suppression in agricultural systems. This review consolidates current knowledge on the mechanisms by which beneficial *Pseudomonas* spp. enhance plant growth. These mechanisms include N fixation, enhancing N fixation in associated diazotrophs, phosphate solubilization, siderophore-mediated iron acquisition, nutrient and niche competitions, antimicrobial metabolite synthesis, and the activation of induced systemic resistance (ISR). Collectively, these mechanisms improve nutrient availability, strengthen plant defense responses, and suppress phytopathogens, thereby supporting sustainable crop production. Despite substantial advances in understanding Pseudomonas–plant interactions, several important challenges remain unresolved. The consistency of Pseudomonas-mediated responses under diverse field conditions is still variable, largely due to differences in soil properties, climatic factors, crop genotype, microbial competition, and agricultural management practices. In addition, the regulatory networks controlling rhizosphere colonization, persistence, metabolic activity, and plant–microbiome interactions remain incompletely understood. Addressing these limitations will be essential for translating mechanistic findings into reliable and scalable agricultural applications. Future research should therefore prioritize integrative and systems-level approaches combining multi-omics and long-term field validation to better elucidate the complex interactions governing *Pseudomonas* functionality in the rhizosphere. Such advances will facilitate the development of robust, site-specific bioinoculants with improved efficacy and consistency across agricultural environments. However, careful biosafety evaluation remains essential, as certain members of the genus possess pathogenic potential. Rigorous strain selection and risk assessment will therefore be critical to ensuring the safe and effective deployment of Pseudomonas-based technologies in sustainable agriculture.

## Data Availability

We do not have any research data outside the submitted manuscript file.
